# Riyadh Mother and Baby Multicenter Cohort Study: The Cohort Profile

**DOI:** 10.1371/journal.pone.0150297

**Published:** 2016-03-03

**Authors:** Hayfaa Wahabi, Amel Fayed, Samia Esmaeil, Rasmieh Alzeidan, Mamoun Elawad, Rabeena Tabassum, Shehnaz Hansoti, Mohie Edein Magzoup, Hanan Al-Kadri, Elham Elsherif, Hazim Al-Mandil, Ghadeer Al-Shaikh, Nasria Zakaria

**Affiliations:** 1 Chair of Evidence Based Health Care and Knowledge Translation, College of Medicine, King Saud University, Riyadh, Saudi Arabia; 2 Department of Community and Family Medicine, King Saud University, Riyadh, Saudi Arabia; 3 Department of Biostatistics, High Institute of Public Health, Alexandria University, Alexandria, Egypt; 4 College of Medicine, Princess Nourah Bint Abdulrahman University, Riyadh, Saudi Arabia; 5 Department of Obstetrics and Gynecology, King Fahad Medical City, Riyadh, Saudi Arabia; 6 Department of Medical Education, College of Medicine, King Saud Bin Abdul-Aziz University for Health Sciences, Riyadh, Saudi Arabia; 7 Department of Obstetrics and Gynecology, King Abdul Aziz Medical City, Riyadh, Saudi Arabia; 8 Department of Obstetrics and Gynecology, King Khaled University Hospital, Riyadh, Saudi Arabia; 9 Department of Medical Informatics and E-Learning Unit, Medical Education Department, King Saud University, Riyadh, Saudi Arabia; King Abdullah International Medical Research Center, SAUDI ARABIA

## Abstract

**Objectives:**

To assess the effects of non-communicable diseases, such as diabetes, hypertension and obesity, on the mother and the infant.

**Methods:**

A multicentre cohort study was conducted in three hospitals in the city of Riyadh in Saudi Arabia. All Saudi women and their babies who delivered in participating hospitals were eligible for recruitment. Data on socio-demographic characteristics in addition to the maternal and neonatal outcomes of pregnancy were collected. The cohort demographic profile was recorded and the prevalence of maternal conditions including gestational diabetes, pre-gestational diabetes, hypertensive disorders in pregnancy and obesity were estimated.

**Findings:**

The total number of women who delivered in participating hospitals during the study period was 16,012 of which 14,568 women participated in the study. The mean age of the participants was 29 ± 5.9 years and over 40% were university graduates. Most of the participants were housewives, 70% were high or middle income and 22% were exposed to secondhand smoke. Of the total cohort, 24% were married to a first cousin. More than 68% of the participants were either overweight or obese. The preterm delivery rate was 9%, while 1.5% of the deliveries were postdate. The stillbirth rate was 13/1000 live birth. The prevalence of gestational diabetes was 24% and that of pre-gestational diabetes was 4.3%. The preeclampsia prevalence was 1.1%. The labour induction rate was 15.5% and the cesarean section rate was 25%.

**Conclusion:**

Pregnant women in Saudi Arabia have a unique demographic profile. The prevalence of obesity and diabetes in pregnancy are among the highest in the world.

## Introduction

Riyadh mother and baby (RAHMA) multicenter cohort study is a hospital-based cohort study conducted in three hospitals in Riyadh, Saudi Arabia. The main aim of the study is to examine the influence of non-communicable diseases such as diabetes, hypertension and obesity, life style changes including urbanization, education and smoking, on the mother, child and the future adult.

Saudi Arabia is a high-income country with a per capita (Gross Domestic Product) GDP of US$ 22,713.40 in 2010. It has a well-developed health care system, which provides services for a population of over 29 million, 70% of whom are Saudi nationals. The total expenditure of the government on health care is 3.2% of the GDP, which amounts to US$ 1,004 per capita [1]. The country has a population growth of 1.49% and a fertility rate of 2.17 child born/woman. The under-25 age group constitutes 51% of the population while the over-65 group constitutes only 3.7% of the population [2].

The rapid urbanization (85% of the population is urban) and changes in the socioeconomic status of the Saudi population have resulted in a significant change in lifestyle, including high consumption of tobacco and fast food and limited physical activity, and the subsequent emergence of non-communicable diseases has become the main public health problem [3]. Population-based studies in Saudi Arabia estimated the prevalence of type 2 diabetes mellitus (T2DM) to be between 21% and 24% [3]. Among Middle Eastern countries, the Gulf region countries showed the highest prevalence of DM; and Saudi Arabia reported the highest prevalence of all [4]. An estimated 28% of the adult female population aged 25 or older has high blood pressure and 43% of those 20 or older are obese [2].

Although Saudi Arabia has a low maternal mortality ratio of 14/100 000, it has a high stillbirth rate of 12/1000 live birth compared to similar high-income countries [2,5]. This could be attributed to the high prevalence of maternal diabetes [6] and obesity [7] in addition to genetic diseases secondary to consanguinity [8]. Due to lack of a national database, however, many maternity health indices are lacking such as the prevalence of gestational diabetes (GDM) and preeclampsia. Most of the data available on the main maternity morbidity indices have been estimated from small hospital-based studies with varying levels of reliability and generalizability [6,9,10]. Because RAHMA has systematically recruited a large number of pregnant women, it is expected to provide accurate estimate of the indices of maternal morbidity in Riyadh and to a great extent in Saudi Arabia generally, bearing in mind that more than 25% of the Kingdom's population (7.3 million) lives in Riyadh [5]. In addition, RAHMA is expected to have a positive influence on the health services and biomedical literature in Saudi Arabia and other Middle Eastern countries by providing hard evidence useful for the refining of policy and practice in maternity services.

RAHMA Executive and Scientific Collaboration Group (see end of paper for membership of these groups) agreed on the following broad objectives:

- To investigate diabetes during pregnancy among pregnant Saudi women, both pre-existing (Pre-GDM) and gestational (GDM), with respect to prevalence, screening methods, management, pregnancy outcomes, postpartum screening and follow up.
- To identify the prevalence of abnormal glucose tolerance in the postpartum period in women who had gestational diabetes and the factors associated with persistent hyperglycemia.
- To investigate the prevalence of different types of hypertensive disorders during pregnancy and their association with maternal micronutrient supplementation.
- To investigate obesity in pregnancy with respect to prevalence, pregnancy outcomes and the existence of programs for management and intervention both during pregnancy and postpartum.
- To investigate the relationship between maternal weight (pre-pregnancy weight and weight gain) in pregnancy and childhood obesity and metabolic syndrome.
- To identify breastfeeding practices in the Saudi community and their relationship to the mother’s education and employment; to identify the relationship between breast feed and postpartum maternal weight loss; and to identify the relationship between breastfeeding and postpartum glycemic profile in mothers who had GDM.
- To determine the most frequently used method of contraception among Saudi women after delivery of a baby and the attitudes and perceptions of pregnant women towards the use of different types of contraception.
- To participate in capacity building for research.

This article, the first in a series of articles addressing the above objectives, describes RAHMA study’s methods and the recruitment phase of the cohort, and provides information on the prevalence, risk factors and maternal and fetal outcomes of these common maternal conditions.

## Objectives

The objectives of this article are to:

1. Describe the design of the RAHMA study.
2. Describe the demographic characteristics of the participants of the study
3. Estimate the prevalence and risk factors associated with GDM, Pre-GDM, hypertensive disorders of pregnancy and maternal obesity.

## Materials and Methods

RAHMA study methods were developed during 2010–2011. In late 2011 to mid-2012, feasibility of the recruitment process and administration of the baseline questionnaire were piloted and assessed. Recruitment of the cohort commenced in the participating hospitals in November 2013 and data collection concluded in March 2015. The participating hospitals were selected by stratified cluster random sampling procedure. The stratifying variable was type of hospital, which included Ministry of Health (MOH), military and university hospitals. Private hospitals were excluded for practical and logistical reasons and because of the small number of deliveries in these hospitals.

Each government hospital in the Riyadh region was identified as being in one of these three strata based on a list obtained from the MOH. Each hospital was considered as a cluster unit. One hospital was then randomly selected from each stratum for a total of three hospitals: King Khalid University Hospital (KKUH), King Fahad Medical City (KFMC) which is run by the MOH, and King Abdul-Aziz Medical City (KAMC), which is a military hospital for National Guards.

### Settings

KKUH is a tertiary referral hospital with 800 beds. It has all the essential departments including 20 operating theaters, an assisted reproduction unit and a cardiac center. The hospital provides free medical care to Saudi nationals and the staff of King Saud University. The obstetrics department provides care for 3000–4000 deliveries per year.

KAMC is a military hospital run by the Ministry of National Guard and is one of the largest medical facilities in the Kingdom; it has 1346 beds, 14 operating theaters and provides both emergency and elective health care in addition to highly specialized services such as liver transplants and assisted reproduction. The obstetrics department provides care for 8000 to 9000 deliveries per year.

KFMC is one of the MOH’s tertiary referral centers. It has 1200 beds, 10 operating theaters and advanced cardiac and oncology centers. The obstetrics department provides care for 4000 to 5000 deliveries per year.

### Eligibility and exclusion criteria

All women who delivered in any of the participating hospitals and their babies were eligible and were invited to participate. Written information about the study was provided to the mothers in Arabic (S1 Text) before consenting. We excluded non-Saudi women from the study.

In Saudi Arabia, antenatal care in the third trimester of pregnancy is provided by obstetric units in hospitals and all deliveries take place in hospitals. Pregnant women can freely change hospitals for antenatal care and for delivery, but since there is no national electronic network of medical records, medical information for women who do change hospitals is sometimes lost. For this study, and to ensure the collection of antenatal, delivery, and newborn data, participants were recruited after delivery in the postnatal ward where they stay for 1–2 days in the case of normal delivery and 3–5 days in the case of cesarean section delivery.

### Sample size

RAHMA is designed to assess the maternal and perinatal outcomes related to common obstetric events that affect Saudi women. To date, no similar studies have been conducted in Saudi Arabia, neither on the same scale nor with a similar scope. To determine the required sample size for RAHMA study, many factors were considered. First, we reviewed international studies such as Born in Bradford [11] and ALSPAC [12], to guide our sample size estimation. Second, the three participating hospitals oversee around 16,000 to 18,000 deliveries per year. From our previous cohort studies, we expected a participation rate of 85%, considering that the study did not involve any intervention or change of treatment plan [13]; we therefore expected to be able to recruit between 13,600 and 15,300 mother/child pairs during the study period. Finally, we considered the power of the study to estimate the prevalence of Pre-GDM, which is one of the main objectives of RAHMA. A minimal sample size was calculated using prevalence of Pre-GDM of 3.7%, as estimated by a previous study [6]. With an error margin of ±0.05, confidence level of 95% and power of 80%, the minimum required sample size to reject the null hypothesis was 14,479 participants.

### Definitions

For purposes of the study, we used the following definitions:

- Due to the variable cut-off values for the diagnosis of GDM and Pre-GDM in the three participating hospitals, we collected the results of the OGTT between 24–34 gestation weeks and fasting blood glucose at ≤ 14 weeks gestation, then re-classified the participants as non-diabetic, Pre-GDM or GDM based on the cut-off values used by the World Health Organization (WHO) [14]
- All participating hospitals follow the same diagnostic criteria for hypertensive disorders of pregnancy based on the report of the American national working group on high blood pressure in pregnancy [15]. In brief, preeclampsia is defined as the new onset of elevated blood pressure after 20 weeks gestation in a previously normotensive woman (≥140 mmHg systolic or ≥90 mmHg diastolic on at least two occasions 6 h apart) in addition to proteinuria of at least 1+ on a urine dipstick or ≥300 mg in a 24-h urine collection [15]. Eclampsia is defined as seizures in a preeclamptic woman that cannot be attributed to other causes [15]. Gestational hypertension is defined as the new onset of elevated blood pressure (≥140 mmHg systolic or ≥90 mmHg diastolic on at least two occasions 6 h apart) after 20 weeks of gestation in a previously normotensive woman. Superimposed preeclampsia is defined as new onset of preeclampsia after 20 weeks of pregnancy on a previously hypertensive woman [15].
- Maternal pre-pregnancy body mass index (BMI) was calculated from maternal recall of weight prior to pregnancy and height measured during the first antenatal clinic. Participants were classified according to the WHO BMI definitions as follows: underweight (<18.5 kg/m^2^), normal weight (18.5–24.9 kg/m^2^), overweight (25.0–29.9 kg/m^2^) and obese (≥ 30 kg/m^2^) [16].
- Maternal pregnancy BMI was calculated from maternal weight at 28–30 weeks gestation and height with the following cut-off values as suggested by Catalano et al [17]: normal weight (≤28.4 kg/m^2^), overweight (28.5–32.9 kg/m^2^) and obese (≥ 33 kg/m^2^) [17].
- Macrosomia is defined as a birth weight of ≥ 4.0 kg.
- Low birth weight (LBW) is defined as a birth weight < 2.5 kg.
- In the three participating hospitals the pregnancy was considered viable after 24 weeks gestation, calculated from the last menstrual period or early ultrasound scan.
- Postdate pregnancy is defined as a pregnancy that continues past 41 completed weeks of gestation [18]
- Preterm birth is defined as birth that takes place before 37 weeks gestation. It is further sub-classified as late preterm (34–36 weeks), moderate preterm (32–33 weeks) and very preterm (<32 weeks’ gestation) [19,20]

### Data collection and management

Each mother/infant pair was allocated a specific identifier for the study, to ensure proper linkage of maternal and child information.

To maximize the use of routinely collected data in the participating hospitals, sources such as laboratory results, mother and newborn medical records and ultrasound reports were accessed. Trained nurses collected maternal antenatal, delivery, and newborn data from medical records (S2 Text). This included data on parity, preterm delivery, mode of delivery, APGAR scores at 5 minutes and stillbirth. Participating mothers completed a self-administered questionnaire on socioeconomic status (S3 Text) including age, education level, working status, family income, marriage to first cousin and tobacco smoking habits.

A research team was established in each of the participating hospitals. The team included midwives and obstetric nurses who were trained to collect data in addition to a general supervisor (head nurse) who ensured that all eligible women were invited to participate in the study and verified data quality by randomly and periodically comparing collected data with its original source. Each research team had a manager who was responsible for the daily operations of the study and for communication between the participating hospital and the study-coordinating center, which was in KAMC. Collected questionnaires and data sheets from each hospital were sent via secured hospital transport to the study-coordinating center on a weekly basis. Data were entered into statistical software as soon as it was received in the coordination center. All files with personal identifying information were locked in a secured place in the coordinating center. Data were backed up into a secured file twice a month.

### Analysis plan

For calculating prevalence of Pre-GDM (type 1 and type 2) and GDM, we included women with:

1. Gestation of 24 weeks or more at the time of delivery, calculated from the last menstrual period and/or early ultrasound scan.
2. Women diagnosed with either T1DM or T2DM before the index pregnancy or meeting the WHO criteria during index pregnancy for the estimation of the prevalence of Pre-GDM as a proportion of the total population with known glycemic status
3. Women with GDM diagnosed according to the WHO criteria during the index pregnancy as a proportion of the total population with known glycemic status.

We excluded women with unknown glycemic status from the analysis.

For calculating prevalence of different hypertensive disorders of pregnancy we estimated the prevalence as a proportion of the total number of women from the cohort with known blood pressure readings during pregnancy.

The prevalence of overweight and obesity was estimated as a proportion of the total cohort by calculating BMI based on self-reported pre-pregnancy weight; a second estimate was calculated based on pregnancy BMI (reported at 28 to 30 weeks gestation).

Demographics of participants in RAHMA cohort were also compared to those of the non-RAHMA cohort (defined as women who delivered during the course of the study but who were not included in RAHMA cohort due to missing data).

### Statistical analysis

Data were analyzed using IBM SPSS for Windows (Version 20.0. Armonk, NY: IBM Corp). Descriptive statistic was used to describe the demographic characteristics of the cohort and the prevalence of different maternal and neonatal outcomes. To compare RAHMA to non- RAHMA cohort, student *t* test was used to compare continuous variables and chi-square test was used to compare categorical variables. A *p* value of < 0.05 was considered statistically significant.

### Ethical statement

The Review Boards of the following institutions reviewed and approved this study:

King Abdullah International Medical Research Centre, approval letter 11/062; King Fahad Medical City Research Centre, approval letter 013–017; and King Saud University, approval letter 13–985. The study was conducted according to the principles expressed in the Declaration of Helsinki. In addition, informed written consent (S1 Text) was obtained from each participant before completing the questionnaire.

## Results

The total number of women who delivered in the three participating hospitals during the study period was 16,019, of which 14,568 women were eligible and consented to participate in the study, 283 women were not eligible (not Saudi nationals), 321 women declined to participate, and 847 women were excluded due to missing data (referred to as non- RAHMA cohort).

The percentages of participants recruited from KKUH, KAMC and KFMC were 95%, 93% and 80% respectively. Demographic characteristics of both RAHMA and non-RAHMA cohorts are shown in (S1 Table).

The demographic characteristics of the RAHMA cohort are shown in Table 1. The mean age of the participants was 29 ± 5.9 years and most had some formal education with over 40% being university graduates. Most of the participants were housewives and 70% were high- or middle-income. Of the total cohort, 24% were married to a first cousin. More than 68%, (95% Confidence Interval (C.I): 67.4–69.5%) of the participants were either overweight or obese when BMI was calculated according to self-reported pre-pregnancy weight. A similar proportion of 69.7%, (95% C.I: 69.0–70.6%) for obesity and overweight was found when BMI was calculated based on pregnancy weight at ≥28 weeks of gestation.

**Table 1:** Maternal Demographic Characteristics for RAHMA Cohort.

Characteristic	RAHMA (N = 14568)
**Age (years)**	29.9±5.9
<20	351(2.4)
20–24	2588 (17.8)
25–29	4406 (30.2)
30–34	3808 (26.1)
35–39	2483(17.0)
≥ 40	932 (6.4)
**Education**	
Illiterate	233 (2.7)
School	4834 (55.7)
University or beyond	3608 (41.6)
Missing	5893
**Working status**	
Housewife	10742 (86.7)
Employed	1552 (12.5)
Student	89 (0.7)
Missing	2185
**Family monthly income per month (SR)**	
Less than 5000 SR	3765 (30.0)
5000–10,000 SR	4769 (38.0)
More than 10,000 SR	4016 (32.0)
Missing	2018
**Pre-pregnancy BMI (kg/m^2^)**	
Underweight < 18.5	167 (2.2)
Normal weight 18.5 to 24.9	2204 (29.4)
Overweight 25 to < 29.9	2466 (32.9)
Obese ≥ 30	2669 (35.6)
Missing	7062
**Pregnancy BMI (kg/m^2^)**	
Normal (≤28.4)	3759 (30.2)
Overweight (28.5–32.9)	3862 (31.0)
Obese (≥33)	4820 (38.7)
Missing	2127
**Parity**	3.6 ± 2.5
Nullipara	3255 (22.4)
2–4	6967 (47.9)
Grand multipara ≥ 5	4328 (29.7)
Missing	18
**Smoking Status**	
Non Smoker	10455 (98.0)
Smoker	209 (1.9)
Missing	3904
**Secondhand Smoking**	
Yes	2618 (22.5)
Missing	2921
**First cousin marriage**	3325 (24)
Missing	715
**Micronutrients Supplements**	
Calcium	10324 (76.1)
Missing	1004
Folic Acid	7650 (56.1)
Missing	929
Iron	11200 (82.2)
Missing	945

Data expressed as means ± SD or N (%). percentages excluding missing data. SR = Saudi Riyal (1 US $ = 3.75SR). RAHMA = **R**iy**a**d**h M**other and B**a**by Multi-centre Cohort Study.

Table 2 shows maternal and neonatal outcomes. The preterm delivery rate was 9%, while 1.5% of the deliveries were postdate. The stillbirth rate was 13/1000 live birth. The prevalence of GDM was 24.2% (95% C.I: 23.4–25.1%) and that of Pre-GDM was 4.3% (95% C.I: 3.9–4.7%). OGTT results were available for 66.8% of the study population and were included in this analysis. OGTT results were missing for the remaining 4845 participants for the following reasons: 2695 participants (18.5%) did not have the test because they were either not booked for antenatal care or booked after 34 weeks of gestation, 1530 participants (10.5%) were screened for GDM during their antenatal care in hospitals other than those participating in the study and the results were not available, and the remaining 582 participants (4%) declined to have the test for various reasons. Comparison of demographic characteristics and determinants of GDM between participants included in the analysis of GDM and those who were not included showed no systematic differences between the two groups in terms of maternal age, parity or BMI (S2 Table). The rate of macrosomia in RAHMA cohort was 3.2%. Of the total RAHMA cohort, 4.0% (95% C.I: 3.7–4.3%) suffered from hypertensive disorders of pregnancy, while the preeclampsia prevalence was 1.2%. No cases of eclampsia were reported. The labour induction rate was 15.5% and the cesarean section rate was 25%.

**Table 2:** Maternal and neonatal outcomes for RAHMA cohort.

Outcome	RAHMA N = 14568
**Pregnancy (per pregnancies)**	
Single	14144 (97.1)
Multiple	424 (2.9)
Missing	_
**Gestational age (for live births)**	
37–41 weeks	12699 (89.5)
34–36 weeks	911 (6.4)
24–33 weeks	361 (2.5)
> 41 weeks	214 (1.5)
Missing	383
**Birth weight (full term) (Kg)**	3.1± 0.45
Average (2.5–3.9)	11788 (90.9)
LBW < 2.5	769 (5.9)
Macrosomia ≥ 4.0	409 (3.2)
Missing	1602
**Gender of infant (M)**	7400 (51.1)
Missing	87
**Living status (Stillbirth)**	184 (1.3)
Missing	415
**Admission to NICU**	704 (4.6)
Missing	137
**Mode of delivery**	
Spontaneous vaginal	10260 (70.9)
Instrumental delivery	593 (4.1)
Caesarean	3612 (25.0)
Missing	103
**Diabetes according to WHO criteria**	
Non-diabetic	6951 (71.5)
GDM	2354 (24.2)
Pre-GDM	418 (4.3)
Missing	4845
**Hypertension disorders**	
Any hypertension	581(4.0)
Pre-existing hypertension	179 (1.2)
Gestational hypertension	235 (1.6)
Preeclampsia/ Superimposed	167 (1.2)
Eclampsia	0.0
Missing	101
**Induction of labor**	2383 (15.5)
Missing	97

Data expressed as mean ± SD or N (%). percentages excluding missing data. RAHMA = **R**iy**a**d**h M**other and B**a**by Multi-center Cohort Study. NICU = Neonatal intensive care unit. LBW = low birth weight. GDM = Gestational diabetes. Pre-GDM = pre-existing diabetes.

## Discussion

RAHMA is the first large cohort study to describe the demographic characteristics and the pregnancy outcomes of the maternity population in Riyadh in Saudi Arabia. While the demographic profile of the cohort is similar in many respects to profiles reported in other high income countries around the world, the prevalence of obesity, diabetes and hypertensive disorders of pregnancy reflect a unique profile of health problems closely associated with changes in lifestyle and the rapid urbanization experienced by residents of the region.

The global burden of adult obesity is recognized in all continents [21]. The prevalence of obesity and overweight among females age 15 or older in Saudi Arabia is reported by WHO to be 65.9%, which is among the highest in Eastern Mediterranean Region countries and is projected to increase to 78% by the year 2022 [22]. Few studies from Saudi Arabia have estimated the prevalence of maternal obesity and its associated adverse outcomes [7,13,23] and risk factors, including urban residence, which increases the risk by almost six fold and high parity, which increases the risk by fivefold [24]. Previous studies which investigated maternal overweight and obesity in Saudi Arabia estimated the prevalence at 44–52% [7,23], which is lower than our finding that 68.3% of the population were either overweight or obese. As women tend to under-report their pre-pregnancy weight [25] we used an additional estimate of maternal weight during pregnancy and included a larger number of the study population We believe our estimate is more precise than previous reports due to the large number of participants and the reporting of two measures of maternal weight i.e. pre-pregnancy weight and weight during pregnancy. Previous reports were either based on a small population [23] or used only mid-trimester pregnancy weight which tends to underestimate the rate of obesity and correlate less accurately with the pregnancy adverse outcomes compared to pre-pregnancy weight [7].

Obesity in pregnancy is a global burden with variable prevalence [26–28]; however, the prevalence we reported in this study is among the highest in the world.

Obesity in pregnancy is a risk factor for many adverse maternal and neonatal outcomes including increased risk of cesarean section delivery, wound infection, macrosomia, and neonatal hypoglycemia [29,30]. Hypertensive disorders of pregnancy and GDM are two of the most serious maternal conditions associated with maternal obesity [29]. Compared to normal-weight pregnant women, those who are obese or over weight have from 1.7 to 4.8 times higher risk of developing hypertensive disorders and 1.7 to 7.4 times higher risk of developing GDM [29].

Based on a recent report by the International Diabetes Federation (IDF), the epidemiology of diabetes during pregnancy is unknown in many countries in the world [31] especially low and middle income countries [32]. However, it is estimated that more than 21 million pregnancies were affected by diabetes in 2013 [31].

Saudi Arabia is one of the top ten countries in the world with the highest prevalence of diabetes [4,31]. A recent household survey reported the prevalence of diabetes among adults age 30 or older to be 25.4%, with 40% of those unaware that they are diabetic [33]. Such a high a burden of the disease in the population is expected to result in a high prevalence of Pre-GDM and GDM during pregnancy [34]. While our finding of GDM prevalence is consistent with the highest rate reported by the IDF (0.4–24.3%) [32], our reported prevalence of 4.3% for Pre-GDM is alarming as it reflects a six-fold increase over the global prevalence of 0.2–0.7% [32].

A few recent hospital-based studies [7,10] as well as a household survey [35] have estimated the prevalence of GDM in the Saudi community to be 15 to 36%. We believe our estimate of 24% is more accurate than the hospital-based studies, which used Carpenter and Cousin’s Criteria for diagnosis of GDM including higher cut-off glucose levels and two or more abnormal values [7,10]. While these criteria were in use for a long time for the diagnosis of GDM, recently, and following studies on hyperglycemia and adverse pregnancy outcomes (HAPO) [36], new criteria were proposed by the International Association of Diabetes and Pregnancy Study Group (IADPSG) [37] and have been endorsed by many international associations including WHO. The main advantage of the new criteria is that they are based on biological effects of hyperglycemia during pregnancy rather than its effects on non-pregnant adults. Furthermore, our estimation of the prevalence of GDM was based on the results of glucose tolerance tests of nearly 10 000 pregnant women; this very large sample size provides better precision compared to the smaller population of pregnant women reported in the household survey [35]. However, it is noteworthy that 33% of the mothers in this study were either not screened for hyperglycemia during pregnancy or the results were not available at the hospital where they delivered. Missing important clinical information such as maternal glycemic status may result in unexpected major neonatal and maternal complications, especially in a community with high prevalence of Pre-GDM and GDM.

Contrary to our expectation of finding a high prevalence of hypertensive disorders in pregnancy, we found the prevalence of any hypertension in pregnancy among the RAHMA cohort to be lower than in many high-income countries [38]. Furthermore, we found the prevalence of preeclampsia in our study population to be lower than that reported for most high and low income countries [38,39].

Many recognized risk factors for preeclampsia were documented in this cohort. GDM, Pre-GDM, advanced maternal age and maternal obesity, which are associated with a two- to three-fold increase in the risk of preeclampsia [40,41], were quite prevalent in the cohort. However, the effects of these factors might have been attenuated by the low rate of other factors observed in this cohort, such as nulliparity, teenage pregnancy and illiteracy, and by the common use of supplemental calcium during pregnancy. Misclassification may explain the low prevalence of preeclampsia in this cohort, especially in the absence of trends or other epidemiological studies from Saudi Arabia. However, it does not explain the observed low prevalence of *all* hypertensive disorders in pregnancy, considering that data were nearly complete and were collected prospectively at the time of delivery. In addition it is highly unlikely that hospital staff would have missed reporting a serious condition such as hypertension during pregnancy.

This contradiction between the high prevalence of pre-eclampsia risk factors and low prevalence of the condition itself has been reported by other investigators [38], which suggests that the epidemiology of risk factors is not the only determinant of preeclampsia; rather, the condition results from the interplay of risk factors and/or the presence of risk factors yet to be discovered.

### Strengths and limitations of the study

The study is the first population-based cohort study in Saudi Arabia specifically and in the Arab countries in general to address obstetric complications and pregnancy outcomes. The results will provide data on the determinants of major health problems and will have a positive influence on the health services and biomedical literature by providing evidence for new policies and practices in maternity services. Furthermore, it highlights gaps in health care services provision in terms of screening and treatment for pregnant women as well as new mothers and their infants.

An important limitation of the study is the large proportion of missing data for some important variables such as participants’ glycemic status, which could have affected the precision of the estimate of Pre-GDM due to smaller than expected sample size. However, lack of precision is unlikely as evident by the narrow confidence interval associated with the prevalence of Pre-GDM. Furthermore, selection bias is unlikely as there were no differences in the key characteristics and the main determinants for diabetes between the two groups (S2 Table). Another limitation is that the study enrolled only Saudi women and their newborns. The non-Saudi population is composed of expatriates who come to Saudi Arabia from many other countries, usually for work; hence, they are a heterogeneous population with diverse determinants of health such as ethnicity and economic status in addition to duration of stay in Saudi Arabia. A final limitation of this study is that biological material was not stored for future analysis due to the limited budget and the lack of research laboratories.

## Conclusion

The RAHMA multicenter cohort study is the first large cohort study to investigate the maternal demographic profile and maternal and neonatal outcomes in Saudi Arabia. Pregnant women in Saudi Arabia have a unique demographic profile. The prevalence of obesity and diabetes during pregnancy are among the highest in the world, which calls for tailored health policies and programs to reduce the prevalence of these conditions and minimize the negative effects on maternal and neonatal health.

## Supporting Information

S1 Table Comparison of demographic characteristics of RAHMA and non-RAHMA cohort. (DOCX)

S2 Table Comparison of the main demographic characteristics and determinants of GDM between women who had OGTT test results and those who did not. (DOCX)

S1 Text Written information and consent form. (DOCX)

S2 Text Data collection sheet. (DOCX)

S3 Text Self-administered questionnaire. (DOCX)
