# Correction: Riyadh Mother and Baby Multicenter Cohort Study: The Cohort Profile

**DOI:** 10.1371/journal.pone.0168420

**Published:** 2016-12-09

**Authors:** Hayfaa Wahabi, Amel Fayed, Samia Esmaeil, Rasmieh Alzeidan, Mamoun Elawad, Rabeena Tabassum, Shehnaz Hansoti, Mohie Edein Magzoup, Hanan Al-Kadri, Elham Elsherif, Hazim Al-Mandil, Ghadeer Al-Shaikh, Nasria Zakaria

The second affiliation for the 11th and 12th authors is not indicated. Hazim Al-Mandil and Ghadeer Al-Shaikh are both affiliated with: Department of Obstetrics and Gynecology, King Khaled University Hospital, Riyadh, Saudi Arabia and College of Medicine, King Saud University, Riyadh, Saudi Arabia.
