# Supplementary material for: Riyadh Mother and Baby Multicenter Cohort Study: The Cohort Profile
Source: PLoS One. 2016 Mar 3;11(3):e0150297. doi: 10.1371/journal.pone.0150297 (PMC4777404; doi:10.1371/journal.pone.0150297)
Supplement: S1 Table — (DOCX) [file pone.0150297.s001.docx]

**S1 Table : Demographic characteristics of RAHMA and non-RAHMA cohorts**

| **Characteristic** | | **RAHMA**  **N=14568** | **Non RAHMA**  **N=847** | **P-value** |
| --- | --- | --- | --- | --- |
| **Age** | | 29.9±5.9 | 29.0±6.0 | <0.01 |
| **Working status** | |  |  |  |
|  | House wife | 10742 (86.7) | 629 (87.8) | 0.51 |
|  | Employed | 1552 (12.5) | 84 (11.7) |  |
|  | Student | 89 (0.7) | 3 (0.4) |  |
|  | Missing | 2185 | 131 |  |
| **Parity** | | 3.6 ±2.5 | 3.4±2.4 | <0.01 |
|  | Nullipara | 3255 (22.4) | 207 (24.5) | 0.1 |
|  | 2-4 | 6967 (47.9) | 413 (48.9) |  |
|  | Grand multipara ≥ 5 | 4328 (29.7) | 224 (26.5) |  |
|  | Missing | 18 | 3 |  |
| **Pregnancy** | |  |  |  |
|  | Single | 14144 (97.1) | 832 (98.2) | 0.05 |
|  | Multiple | 424 (2.9) | 15 (1.8) |  |
| **Gestational age (for live births)** | |  |  |  |
|  | 37 - 41 weeks | 12699 (89.5) | 677 (86.2) | <0.01 |
|  | 34-36 weeks | 911 (6.4) | 58 (7.4) |  |
|  | 24-33weeks | 361 (2.5) | 33 (4.2) |  |
|  | More than 41 weeks | 214 (1.5) | 17 (2.2) |  |
|  | Missing | - | 2 |  |
| **Birth weight (full term)** | |  |  |  |
|  | Average (2.5-3.9) Kg | 11788 (90.9) | 637 (91.7) | 0.8 |
|  | LBW < 2.5 Kg | 769 (5.9) | 38 (5.5) |  |
|  | Macrosomia > 4.0 Kg | 409 (3.2) | 20 (2.9) |  |
| **Gender of infant (M)** | | 7400 (51.1) | 426 (50.9) | 0.9 |
| **Living status (Stillbirth)** | | 184 (1.3) | 24 (2.8) | <0.01 |
| **Mode of delivery** | |  |  |  |
|  | Vaginal delivery | 10260 (70.9) | 655 (78.4) | <0.01 |
|  | Instrumental delivery | 593 (4.1) | 30 (5.1) |  |
|  | Cesarean section | 3612 (25.0) | 150 (17.9) |  |
|  | Missing | 103 | 12 |  |

Data expressed as mean ± SD or N (%). Percentages excluded missing data.

RAHMA= **R**iy**a**d**h** **M**other and B**a**by Multi-center Cohort Study
