# Supplementary material for: Riyadh Mother and Baby Multicenter Cohort Study: The Cohort Profile
Source: PLoS One. 2016 Mar 3;11(3):e0150297. doi: 10.1371/journal.pone.0150297 (PMC4777404; doi:10.1371/journal.pone.0150297)
Supplement: S2 Table — (DOCX) [file pone.0150297.s002.docx]

Comparison of the main demographic characteristics and determinants of GDM between women who had OGTT test results and those who did not showed no systematic difference between the two groups

| Characteristic | Women with data available for glycemic classification  Mean± SD | Women with missing data for glycemic classification  Mean±SD |
| --- | --- | --- |
| Mother's Age( years) | 30.2±5.9 | 29.3±5.9 |
| Number of pregnancy | 3.7±2.5 | 3.4±2.4 |
| Number of deliveries | 2.3±2.1 | 2.2±2.1 |
| Mother's weight on delivery (Kg) | 78.7±14.8 | 77.9±14.6 |
| Gestational Age at Delivery (weeks) | 38.6±2.2 | 38.4±2.5 |
| Pregnancy BMI (kg/m^2^) | 31.8±5.8 | 31.4±5.6 |
| Pre-pregnancy BMI (kg/m^2^) | 28.4±5.6 | 27.8±5.5 |
