# Supplementary material for: Riyadh Mother and Baby Multicenter Cohort Study: The Cohort Profile
Source: PLoS One. 2016 Mar 3;11(3):e0150297. doi: 10.1371/journal.pone.0150297 (PMC4777404; doi:10.1371/journal.pone.0150297)
Supplement: S1 Text — (DOCX) [file pone.0150297.s003.docx]

**أمهات ومواليد مدينة الرياض: دراسة متعددة المراكز**

معلومات عن الدراسة:

هي دراسة طويلة المدى لمجوعة من الامهات ومواليدهن في ثلاث مشافي في مدينة الرياض. تختص الدراسة بإحصاء الامراض المصاحبة للحمل كمرض السكري وسكري الحمل وتأثيرها على صحة الجنين والام وذلك بمقارنة نتاج الحمل للأمهات المصابات وغير المصابات. كما سيتم تعقب هؤلاء الأطفال من فترة الحمل وخلال مرحلة الطفولة وحتى سن البلوغ. هذه الدراسة هي وسيلة قوية تساعد في البحث عن العديد من العوامل التي تأثر على صحة المجتمع، وتأثير البيئة ونوعية الغذاء على صحة الاجيال القادمة. كما سيتم استخدام المعلومات التي تم جمعها في هذه الدراسة للتعرف على الأسباب الشائعة لأمراض الطفولة واستكشاف النمو العقلي والاجتماعي لهذا الجيل الجديد.

**نموذج الموافقة**

**سيتم الاحتفاظ بنسخة من هذا النموذج في سجللك الخاص في المستشفى**

أؤكد أنني قد قرأت وفهمت ورقة المعلومات بتاريخ ................... وأتيحت لي الفرصة لطرح الأسئلة و فهمت أيضا أن مشاركتي أنا وطفلي طوعية وأن لنا كامل الحرية في الأنسحاب في أي وقت دون إبداء أي سبب ودون أن تتأثر حقوقنا في الرعاية الطبية.
وأنا أفهم أن الباحثين العاملين في هذه الدراسة سيطلعون على السجلات الطبية الخاصة بي وبطفلي قد يقوم باحثين آخرين لهم صلة بالدراسة بالاطلاع أيضا على السجلات الطبية وذلك بعد موافقة اللجنة التنفيذي لأخلاقيات البحوث.
أنا أوافق على أن أحد الباحثين بالدراسة يمكنه الاتصال بي في المستقبل باستخدام رقم ا لهاتف أو عنوان البريد الإلكتروني الخاص بي.

أنا أوافق على إعطاء العينات البيولوجية، والتي قد تشمل الدم واللعاب والبول لاستخدامها في الدراسة المذكورة أعلاه والتي يمكن تخزينها لاستخدامها في المستقبل.

**إسم المشاركه : ................... التوقيع ........ التاريخ:......................**

**إسم الباحث : ................... التوقيع ........ التاريخ:......................**
