# Supplementary material for: Riyadh Mother and Baby Multicenter Cohort Study: The Cohort Profile
Source: PLoS One. 2016 Mar 3;11(3):e0150297. doi: 10.1371/journal.pone.0150297 (PMC4777404; doi:10.1371/journal.pone.0150297)
Supplement: S2 Text — (DOCX) [file pone.0150297.s004.docx]

**Riyadh Mother and Baby Multicenter Cohort Study**

1. Medical Record number: Mother age: Hospital:
2. Nationality ם Saudi ם Non-Saudi
3. This pregnancy ם Singleton ם Multiple

…………………………………………………………………………………………………

**Mother Demographic information**

1. Gravidity:………………
2. Parity:…………….
3. Number of miscarriage :……………..

1. Last menstrual period :……./……/……dd/mm/yy ם Not Known
2. Expected date of delivery by ultrasound scan : …./…/……dd/mm/yy ם Not Known
3. Gestational age at delivery:………………..
4. Was an anomaly scan performed? ם Yes ם No
5. Actual date of delivery :……./……/……dd/mm/yy

………………………………………………………………………………………………

**Pre-pregnancy care and antenatal care**

1. Was the mother taking Folic Acid before she was pregnant םYes ם No ם Not Known
2. Gestation age at the 1st antenatal visit…………….. ם Not Known ם No antenatal care
3. What was the HBA1c at booking ………… ם Not done ם Not applicable
4. What was the HBAIc 3^rd^ trimester…………. ם Not done ם Not applicable
5. Maternal Height at booking:…………… םUnknown
6. Maternal Weight at booking:…………….. םUnknown
7. Diabetes status:………………..

ם Non-diabetic ם Type 1 ם or Type 2 ם Gestational diabetes םUnknown

1. Diabetes treated by ם diet ם insulin םdiet& insulin םnot applicable
2. Was the mother screened for hyperglycemia at 1^st^ trimester : ם Yes ם No
3. What is the screening method? ם fasting blood glucose םOGTT ם Not screened at all
4. Was the mother screened for gestational diabetes between 24-32wks? ם Yes ם No
5. Did the mother have any of the following pregnancy complications:

- Pre-existing Hypertension ם Yes ם No
- Pregnancy induced hypertension ם Yes ם No
- Pre-eclampsia ם Yes ם No
- Intera-uterine growth restriction ם Yes ם No
- Polyhydramnios ם Yes ם No
- Antenatal fetal distress. ם Yes ם No

1. Did the mother have any of the delivery complications:

- Shoulder dystocia ם Yes ם No
- Fetal trauma ם Yes ם No
- Third /fourth degree tear ם Yes ם No

………………………………………………………………………………………………

**Maternal Outcomes**

1. Induced labour ם Yes ם No
2. Rout of delivery

- Spontaneous vaginal delivery
- Instrumental delivery
- Elective cesarean section
- Emergency cesarean Section

1. Maternal admission to intensive care unit for any reason ם Yes ם No
2. Maternal death ם Yes ם No

………………………………………………………………………………………………

ם

**Neonatal Outcomes**

Baby medical record number:…………………………

1. Living status : ם liveborn ם Fresh stillbirth ם Macerated stillbirth
2. New born gender ם Female ם Male
3. APGAR at 5min ……………
4. Birth weight (Kg)………………
5. Congenital malformation diagnosed : ם Yes ם No

Description of malformation:

- Cardiac anomaly ם Yes ם No
- Renal anomaly ם Yes ם No
- Central nervous system anomaly ם Yes ם No
- Musculoskeletal anomaly ם Yes ם No
- Other anomaly ם Yes ם No

1. Neonatal admission to neonatal intensive care unit ם Yes ם No
