# Supplementary material for: Riyadh Mother and Baby Multicenter Cohort Study: The Cohort Profile
Source: PLoS One. 2016 Mar 3;11(3):e0150297. doi: 10.1371/journal.pone.0150297 (PMC4777404; doi:10.1371/journal.pone.0150297)
Supplement: S3 Text — (DOCX) [file pone.0150297.s005.docx]

**Riyadh Mother and Baby Multicenter Cohort Study**

**Socio-economic determinants of health**

1. Are you related to the father of your baby other than by marriage? For example are you cousins?

Yes No Don't Know

1. If yes, how are you related to the father of your baby? e.g. 1st cousin, 2nd cousin (Cross ONE box ONLY)

1st Cousin Second Cousin Other related by marriage Don’t know

1. Were your parents related? For example were they cousins? (Cross ONE box ONLY)

Yes No Don't Know

1. If yes, how are your parent related to each other? e.g. 1st cousin, 2nd cousin (Cross ONE box ONLY)

1st Cousin Second Cousin Other related by marriage Don’t know

1. Were the parents of the father of your baby related? For example were they cousins? (Cross ONE box ONLY)

Yes No Don't Know

1. If yes, how were they related? (Cross ONE box ONLY)

1st Cousin Second Cousin Other related by marriage Don’t know

The family monthly income in Saudi Riyals:

- Less than 3000
- 3000-4999
- 4999-5000
- 5000-6999
- 7000-9999
- 10000-14999
- 15000-19000
- 20000 or more
- Not specified
- Refused to answer question

**Mother education**

- Illiterate
- Primary school
- Secondary school
- High school
- College or above

**Father education:**

- Illiterate
- Primary school
- Secondary school
- High school
- College or above

**Mother’s work**: Yes/No

If (Yes) what work:

Government private sector self-employed (her own work)

How many hours does the father work per day

**Father’s work** Yes/No

If (Yes) what work:

Government private sector self-employed (his own work)

How many hours does the father work per day

**Housing:**

1. Do you live in rented accommodation Yes/No
2. Do you live in your own property Yes/No
3. Do you live in your parent or in-laws accommodation Yes/No
4. How many rooms are in the accommodation
5. How many adults live in the accommodation (above 18 years) including yourself
6. How many children live in the accommodation (below 18 years)
7. How many elderly live in the accommodation (65 years and above)

**Are you**

- Married (first marriage)
- Re-married
- Divorced
- Widowed

**Smoking:**

1. Have you ever regularly smoked cigarettes; that is at least one cigarette a

day? (Cross ONE box ONLY)

Yes for more than 1 year Yes for less than 1 year No

1. If yes how old were you when you started smoking cigarettes?

Age: Years old Don't Remember

1. Were you smoking during your last pregnancy Yes/No
2. How many cigarettes do/did you smoke during pregnancy, or in the three months before pregnancy? (Cross ONE box ONLY in each row)

a) 3 months before pregnancy non 1-5 6-10 11-20 20+

b) First 3 months of pregnancy non 1-5 6-10 11-20 20+

c) Since the beginning of 4th month non 1-5 6-10 11-20 20+

1. During pregnancy have you been exposed to other peoples' cigarette smoke at work or at home Yes/No
2. If Yes, for how many hours per day approximately? (Cross ONE box ONLY )

Less than 1 hour per day/occasionally more than 1hour (specify number)

1. Have you used any other tobacco products like sheesha during pregnancy, or in the 3 months before pregnancy?

Yes No Don't Know

1. If yes how many times a week

Occasionally regular (specify times per week)
